# Role of Group I Metabotropic Glutamate Receptors in Spike Timing-Dependent Plasticity

**DOI:** 10.3390/ijms23147807

**Published:** 2022-07-15

**Authors:** Irene Martínez-Gallego, Antonio Rodríguez-Moreno, Yuniesky Andrade-Talavera

**Affiliations:** Laboratory of Cellular Neuroscience and Plasticity, Department of Physiology, Anatomy and Cell Biology, Universidad Pablo de Olavide, ES-41013 Seville, Spain; imargal@upo.es

**Keywords:** STDP, glutamate receptor, mGluR, timing, synaptic plasticity

## Abstract

Metabotropic glutamate receptors (mGluRs) are G-protein-coupled receptors that exhibit enormous diversity in their expression patterns, sequence homology, pharmacology, biophysical properties and signaling pathways in the brain. In general, mGluRs modulate different traits of neuronal physiology, including excitability and plasticity processes. Particularly, group I mGluRs located at the pre- or postsynaptic compartments are involved in spike timing-dependent plasticity (STDP) at hippocampal and neocortical synapses. Their roles of participating in the underlying mechanisms for detection of activity coincidence in STDP induction are debated, and diverse findings support models involving mGluRs in STDP forms in which NMDARs do not operate as classical postsynaptic coincidence detectors. Here, we briefly review the involvement of group I mGluRs in STDP and their possible role as coincidence detectors.

## 1. Introduction

Glutamate is the major excitatory neurotransmitter of the central nervous system, and its actions are mediated by the activation of a diverse family of receptors that can be divided into two large sets comprising ionotropic glutamate receptors (the α-amino-3-hydroxy-5-methyl-4-isoxazole propionate (AMPA)-, N-methyl-D-aspartate (NMDA)- and kainate (KA)-type receptors) and metabotropic glutamate receptors (mGluRs). Although the fast glutamate excitatory synaptic transmission is typically mediated by the ionotropic ligand-gated glutamate receptors, the slower and long-lasting effects of glutamate are generally mediated by mGluRs [1,2]. mGluRs are G-protein-coupled receptors (GPCRs) that share common topology and exhibit enormous diversity in their expression patterns, sequence homology, pharmacology, biophysical properties and signaling pathways among the different receptor’s subtypes [2,3].

Based on these properties, mGluRs can be divided into three groups: group I, II and III (mGluR I, mGluR II and mGluR III, respectively). mGluR I includes mGluR1 and mGluR5 receptors that are positively coupled to phospholipase C (PLC), whereas mGluR II (comprising mGluR2 and mGluR3) and mGluR III (including mGluR4 and mGluR6-8) are negatively coupled to the formation of adenylate cyclase-mediated cAMP [4]. The metabotropic nature of mGluR signaling was first discovered by the demonstration that glutamate could stimulate the formation of inositol trisphosphate (IP_3_) production, thus showing that glutamate, similar to other neurotransmitters such as acetylcholine, could also trigger intracellular pathways by activating G protein-coupled receptors [5].

mGluRs modulate different aspects of neuronal physiology, particularly, excitability and plasticity [1,4,6]. They are widely expressed throughout the brain and are found at both pre- and postsynaptic sites of excitatory glutamatergic synapses. Their location makes mGluRs ideally suited to modulate diverse processes and mechanisms of synaptic transmission and plasticity (e.g., glutamate release and intracellular pathways underlying postsynaptic forms of plasticity). Synaptic plasticity has been widely accepted as a possible functional substrate for memory encoding, information processing and neuronal circuit refinement during development [7]. In a classical view, repetitive activation of synapses drives synaptic changes that entail long-term potentiation (LTP) or long-term depression (LTD) of the synaptic transmission. These forms of long-term changes of synaptic strength studied in ex vivo and in vivo preparations depend on the pattern of stimulation used [8,9,10,11,12,13]. For instance, high-frequency stimulation-induced LTP (HFS-LTP) has been intensively studied and it is known to be NMDAR-dependent [5,14].

Under certain conditions, mGluRs can serve as co-triggers for the induction of NMDAR-dependent LTP [15,16], possibly by facilitating the activation of NMDARs [6,17]. mGluR II is well known to be involved in LTD in the hippocampus [18,19,20] and mGluR III has also been involved in plasticity [21,22]. Depending on the brain region, postsynaptic cell type, and specific intracellular pathways, mGluR I is particularly known for inducing LTD, which can be mediated by either mGluR1 or mGluR5 [23,24]. In addition to their role in LTD, the activation of mGluR I potentiates NMDA-receptor-mediated currents [25,26], and it can also depolarize several types of neurons through activation of a Ca^2+^-dependent cation conductance [27,28].

Some conceptual controversies exist regarding the role of mGluR I and the type of synaptic plasticity it involves (see Jones, 2017; [29] for review). Notably, this group of metabotropic receptors was proposed as a crucial player in the mechanisms underlying the detection of activity coincidence in the form of synaptic plasticity that depends on the precise temporal coincidence and order of pre- and postsynaptic activities: the spike timing-dependent plasticity (STDP) [30,31,32]. STDP has been found in all of the species in which it has been studied (from insects to humans) [33]. Different forms of STDP have been described depending on the specific cell and synapse type [34,35,36,37,38,39,40], the state of the network [41], neuromodulatory agents [42,43] and the developmental stage [44,45] in which the study was carried out, and it is believed that STDP endows a supporting role to memory formation and maintenance [9,30]. Here, we review and discuss the involvement of mGluR I in STDP and its role as a coincidence detector.

## 2. The STDP Phenomena and mGluRs Involvement

**Figure 1:**
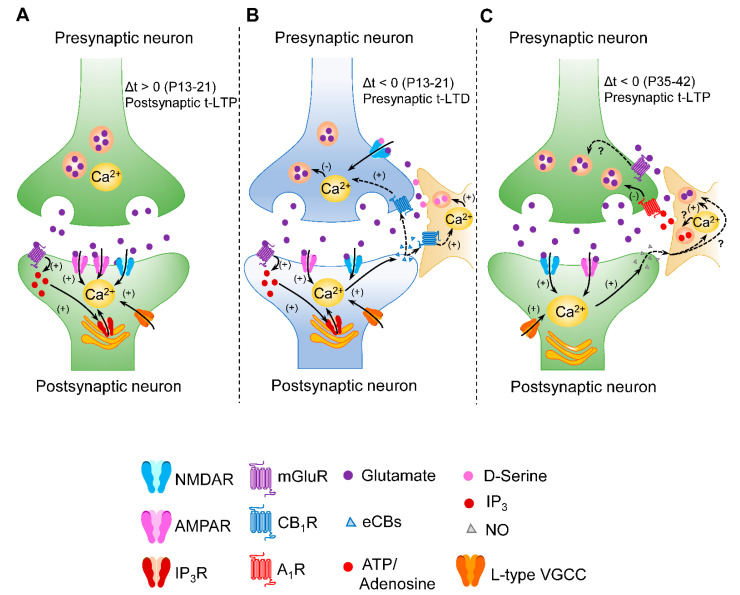
Schematic summarizing the role of mGluR I in different forms of STDP, showing that two different large sets of STDP forms could be proposed according to the underlying mechanism for coincidence detection: (**A**) In a classic model of Hebbian STDP, postsynaptic NMDARs are the main coincidence detectors (providing strong and brief Ca^2+^ signals) that drive postsynaptic forms of t-LTP and could also involve postsynaptic VGCCs, postsynaptic mGluRs and IP3R-mediated increase in postsynaptic Ca^2+^ [30,33,34,47,50]. This model does not fully support the mechanism underlying postsynaptic NMDAR-dependent t-LTD at horizontal layer 2/3-layer 2/3 synapses of the primary somatosensory cortex [36]. Consequently, more research needs to be performed. In turn, other findings support the proposed model involving the mGluR-VGCC-IP3R pathway in presynaptic forms of t-LTD as shown in B and t-LTP as shown in C as representative examples. In these studies, presynaptic forms of STDP either involve non-postsynaptic and likely presynaptic NMDARs and eCBs as retrograde signal driving to t-LTD, as represented in B, [34,47] or NO retrograde signal driving to NMDAR-independent t-LTP, as represented in C [44]. In addition, astrocytes release D-serine for t-LTD at P13-P21 (**B**) and ATP/adenosine for t-LTP at P35-42 (**C**). The presence and involvement of astrocytic mGluRs in diverse forms of STDP should not be discarded and deserve further experimental efforts. Note that at hippocampal area CA1, a developmental switch occurs: when the postsynaptic activity precedes the presynaptic activity (∆t < 0) at P13-21, presynaptic t-LTD is induced (**B**) whereas presynaptic t-LTP is induced by the same protocol at older ages (P35-P42) (**C**) with astrocytes commanding the closing and opening of these plasticity windows [44,46]. Such general models represented in (**A**–**C**) are constantly evolving and, therefore, must be revisited considering future advances. (-): decrease in glutamate release in the presynaptic form of t-LTD, (+): pathways that are activated, (?): mechanistic insights that are yet to be demonstrated.

STDP is a form of synaptic plasticity in which the coincidence of pre- and postsynaptic activities (spiking) within a few milliseconds dictates a long-lasting potentiation (t-LTP) or depression (t-LTD) of synaptic transmission depending on the order of the spiking occurrence. In excitatory synapses, when the presynaptic firing precedes the postsynaptic spiking (“pre-post”), t-LTP is induced, whereas when the order is the inverse (“post-pre”), t-LTD is induced [30,33] (Figure 1). However, there are exceptions according to the recent advances in the field (i.e., post-pre protocol induces t-LTP in the hippocampal area CA1 of mice at postnatal days 35–42 (P35-P42) [44], Figure 1B) (see also Feldman, 2012; [30] for review).

Different forms of STDP were found in diverse brain areas, including the hippocampus [34,44,46], diverse cortical synapses [36,45,47,48,49], the cerebellum [38], the spinal cord [50], the striatum [37] and the amygdala [51] among others, and mGluR I is involved in t-LTP and t-LTD at different synapses in some of the these regions, covering different functions. Thus, in the somatosensory cortex, postsynaptic mGluR I participates in the production and release of eCB, which acts as a retrograde signaling molecule [47]; in neocortical synapses onto interneurons, where it participates in t-LTD [52]; in the *substantia gelatinosa*, it regulates the polarity of STDP [50] and in corticostriatal synapses, it acts as a coincidence detector for t-LTD [37]. In the cerebellum, mGluR I participates in the induction of t-LTP but not t-LTD [38]. Recently, it has been observed that eyeblink conditioning, a form of Pavlovian learning that engages discrete areas of the cerebellar cortex and deep cerebellar nuclei, is impaired in mGluR1 knockout mice. Moreover, administration of the mGluR1/5 agonist DHPG into the *lobulus simplex* region of the cerebellar cortex promotes eyeblink conditioning in rats, which indicates that cerebellar mGluR1 plays a role in cerebellar-dependent associative learning [53]. mGluRs have been also found to be involved in STDP in the hippocampus, where they seem to gate NMDAR-mediated t-LTP [54], participate postsynaptically in the induction of a presynaptic form of t-LTD by promoting the production and release of eCB that acts as a retrograde signaling molecule [34,44] or presynaptically participate in a newly discovered form of presynaptic t-LTD [44]. Whether t-LTD and t-LTP occur at the same time on the same synapse, the underlying pathways involved in their expression remain to be fully determined. Additionally, whether these forms of STDP share the same or distinct pools of Ca^2+^ in the same dendritic spine is still puzzling and deserves further research.

## 3. Group I mGluR Involvement in the Coincidence Detection for STDP

A mechanism for detection of the pre- and postsynaptic activities and their correlated timings appears crucial for codifying the spiking coincidence that drives STDP [30,33]. In a classical view, postsynaptic NMDARs are the receptors acting as coincidence detectors [5,6,30] (Figure 1A) and their contribution to STDP has been largely documented from either pre- or postsynaptic synaptic compartments [31,33,34,39,47,48,49]. However, presynaptically located NMDARs do not likely hold the role of coincidence detectors in NMDAR-dependent forms of STDP [55]. This evidence suggests the existence of different mechanisms of STDP that are mediated by different coincidence detectors [34,36,39,40,47,56,57,58,59,60] (Figure 1). Therefore, two different heterogeneous classes of STDP could be proposed: one class that involves NMDARs as the primary coincidence detectors [34,36,39,40,47,56,57,58,59] (Figure 1A) and another class that does not require NMDARs, or they do not codify the coincidence [34,39,40,44,46,47,60,61,62,63], and includes pre- or postsynaptic mGluRs (Figure 1B,C). Accordingly, irrespective of NMDAR involvement, as previously mentioned, it is known that STDP requires other players including mGluRs and retrograde messengers such as endocannabinoids (eCBs) or NO and involve astrocytes [34,39,40,44,46,47,60,61,62,63] (Figure 1B,C). Moreover, mGluRs could be proposed as relevant players in the coincidence detection mechanisms for STDP where NMDARs are not involved in such mechanisms.

In this regard, unlike ionotropic glutamate receptors, mGluRs are thought to operate within a larger timescale [6]. Thus, it turns out to be more intriguing how mGluRs can efficiently contribute as detectors of coincident activity that takes place in a time window of a few milliseconds (i.e., 5–10 ms) during few repetitive pairings (i.e., <100 times) as it happens for STDP [30,33]. However, even though GPCRs such as mGluRs are supposed to signal over a timescale of seconds to minutes, mGluR signaling has been suggested to also occur faster and with a timescale similar to that observed for ionotropic glutamate receptors [6]. This notion is supported by conformational studies showing large-scale, activation-associated mGluR1 inter-subunit changes on a millisecond timescale [64].

Consequently, most of the coincidence detector models propose mGluR-voltage-gated Ca^2+^ channel (VGCC)-IP_3_R signaling as a principal postsynaptic coincidence detector. In addition, phospholipase C (PLC) and IP_3_Rs have been proposed for serving well as molecular coincidence detectors. This pathway entails a strong Ca^2+^ dependence that can synergistically contribute to eCB signaling, thus driving some forms of t-LTD [30,47,65]. In fact, these models find support in several forms of STDP that depend on changes in the cytosolic Ca^2+^ dynamics involving Ca^2+^ influx through VGCCs and/or Ca^2+^ mobilization from internal stores [34,37,38,44,47,50,52,66,67,68,69] (Figure 1). Moreover, there is evidence that in the spine machinery, the release of Ca^2+^ from internal stores and Ca^2+^ transients through VGCCs are likely to provide highly localized and input-specific Ca^2+^ signals to induce synaptic plasticity [65,67].

Particularly, Ca^2+^ from VGCCs considerably facilitates mGluR-dependent PLC activation, acting independently as a co-agonist of IP_3_Rs to promote IP3-dependent Ca^2+^ release [70]. Thus, in this model, a Ca^2+^ influx through VGCCs during each postsynaptic action potential (spike) could transiently trigger mGluR-IP_3_ signaling [67,71,72]. This could provide an adequate timing for the presynaptic spiking to drive mGluR signaling and release enough Ca^2+^ to trigger Ca^2+^-dependent eCB synthesis and release that ultimately leads to LTD. A similar mechanism has been proposed for short-term synaptic depression with the involvement of VGCCs and mGluR I activation that synergistically drives eCB release [70,73]. This model appears essentially the same as the two-coincidence detector model for STDP that was previously proposed by Karmarkar and Buonomano (2002). Additionally, small increases of IP_3_, which are not sufficient to stimulate release directly, can enhance the Ca^2+^ sensitivity of the IP_3_Rs, thereby transforming the cytoplasm into an excitable medium able to produce Ca^2+^ waves [65,74]. For example, inhibiting the hydrolysis of IP_3_ greatly enhances the sensitivity of neurons to synaptic stimulation [65].

As indicated above, mGluR I also mediates a form of cortical t-LTD that is independent of postsynaptic NMDARs and is presynaptically expressed [47]. This form of t-LTD, induced at layer 4 to layer 2/3 synapses in the primary somatosensory cortex, involves postsynaptic mGluRs and retrograde eCB signaling, suggesting a common signaling motif that is in line with the proposed roles of mGluR I in the models of coincidence detection. More recently, in the hippocampus, a presynaptic form of hippocampal t-LTD shows similar properties to that previously described in the neocortical synapses during development (12-18 postnatal days). This form of t-LTD is presynaptic, requires postsynaptic mGluR5, eCB type 1 receptors (CB_1_R), postsynaptic Ca^2+^, astrocytic signaling (D-serine release) and non-postsynaptic NMDA receptors at Schaffer collateral-CA1 synapses [34]. Hence, the described mechanism matches with the above-mentioned class of STDP (Figure 1A).

Notably, this form of hippocampal t-LTD switches from depression to potentiation (t-LTP) across a wide range of spike timings as young mice mature towards the fifth postnatal week [44]. Interestingly, this form of t-LTP is also expressed presynaptically and requires the activation of mGluR5, but not NMDARs. In addition, the required activation of mGluR5 appears to reside presynaptically. At these synapses, presynaptic mGluRs have been described to bidirectionally modulate glutamate release [75]. Moreover, glial cells are also thought to express mGluRs that probably contribute to the influence of synaptic plasticity [76,77]. In turn, mGluRs located in the astrocytes appeared not to be involved in this particular form of t-LTP.

## 4. Concluding Remarks

Cooperative and correlated activity within neuronal circuits underlie information processing through the formation of neuronal network ensembles and short- and long-lasting plastic changes that are associated with and/or underlie higher cognitive processes such as memory formation and recall [13,78,79]. In the case of STDP, cumulative evidence suggests that either pre- or postsynaptic mGluR I may drive t-LTP and t-LTD, acting as a key component of non-classical postsynaptic NMDAR-based detection of activity coincidence. Thus, group I mGluRs have emerged as potent modulators/drivers of significant aspects of neuronal circuit functioning including STDP. Additionally, growing evidence posits mGluRs as suitable drug targets to treat neurological disorders such as anxiety, Parkinson’s disease, autism spectrum disorders, Alzheimer’s disease, fragile X syndrome and drug abuse [4,29,80,81] where STDP has been or could be found impaired. However, despite the great advances in studying the functional role of mGluRs in neuronal circuits and, in particular, in STDP, more research is needed to cover missing mechanistic insights that probably depend on the current experimental and technical limitations. In this regard, a direct demonstration of the functional presence of mGluRs at the presynaptic compartment could be achieved by paired recordings of connected pyramidal neurons while blocking the metabotropic signaling just in the presynaptic neuron, as it was previously performed to demonstrate the functional presence of NMDARs driving t-LTD in layer 4–layer 2/3 of the mouse barrel cortex [39,56]. As well, the occurrence of t-LTD and t-LTP at the same synapse involving presynaptic NMDARs, mGluRs and mGluRs located in the astrocytes could be unveiled by performing the above-mentioned arduous approach. Development of caged mGluR I antagonists that could be uncaged locally would allow for specific determinations of mGluR involvement in STDP at cellular and subcellular compartments.
